# Rapid Urban Malaria Appraisal (RUMA) III: epidemiology of urban malaria in the municipality of Yopougon (Abidjan)

**DOI:** 10.1186/1475-2875-5-29

**Published:** 2006-04-04

**Authors:** Shr-Jie Wang, Christian Lengeler, Thomas A Smith, Penelope Vounatsou, Guéladio Cissé, Marcel Tanner

**Affiliations:** 1 Swiss Tropical Institute (STI), P.O. Box, CH-4002 Basel, Switzerland; 2 Centre Suisse de Recherches Scientifiques (CSRS), 01 B.P. 1303 Abidjan, 01 Ivory Coast

## Abstract

**Background:**

Currently, there is a significant lack of knowledge concerning urban malaria patterns in general and in Abidjan in particular. The prevalence of malaria, its distribution in the city and the fractions of fevers attributable to malaria in the health facilities have not been previously investigated.

**Methods:**

A health facility-based survey and health care system evaluation was carried out in a peripheral municipality of Abidjan (Yopougon) during the rainy season of 2002, applying a standardized Rapid Urban Malaria Appraisal (RUMA) methodology.

**Results:**

According to national statistics, approximately 240,000 malaria cases (both clinical cases and laboratory confirmed cases) were reported by health facilities in the whole of Abidjan in 2001. They accounted for 40% of all consultations. In the health facilities of the Yopougon municipality, the malaria infection rates in fever cases for different age groups were 22.1% (under one year-olds), 42.8% (one to five years-olds), 42.0% (> five to 15 years-olds) and 26.8% (over 15 years-olds), while those in the control group were 13.0%. 26.7%, 21.8% and 14.6%, respectively. The fractions of malaria-attributable fever were 0.12, 0.22, 0.27 and 0.13 in the same age groups. Parasitaemia was homogenously detected in different areas of Yopougon. Among all children, 10.1% used a mosquito net (treated or not) the night before the survey and this was protective (OR = 0.52, 95% CI 0.29–0.97). Travel to rural areas within the last three months was frequent (31% of all respondents) and associated with a malaria infection (OR = 1.75, 95% CI 1.25–2.45).

**Conclusion:**

Rapid urbanization has changed malaria epidemiology in Abidjan and endemicity was found to be moderate in Yopougon. Routine health statistics are not fully reliable to assess the burden of disease, and the low level of the fractions of malaria-attributable fevers indicated substantial over-treatment of malaria.

## Introduction

During the last two decades, African countries have experienced rapid urban growth without a corresponding development of urban infrastructure and services. This has profoundly changed environmental and disease patterns. Recently there has been a growing interest in the study of urban malaria epidemiology, with the aim of developing specific control strategies [1-4].

In 2000, approximately 7.4 million people or 45.8% of the total population of Côte d'Ivoire lived in urban areas, thus the country was considered highly urbanized [5,6]. In 1990, 15–17% of the population lived in informal settlements and among them, 60% lived in slums with poor road and sanitation infrastructure [7], as the local government finds it difficult to cope with the most urgent demands of this uncontrolled growth.

A retrospective study from 1985 to 1998 in the unit of infectious diseases of the Centre Hospitalier Universitaire (CHU) of Treichville showed that 274 cases of severe malaria (0.5%) were detected among 54,098 hospitalizations [8]. The records in the paediatric department of the CHU of Yopougon from January, 1998 to December, 2001 showed that 57.2% of children diagnosed as severe malaria had anaemia, and 55% of them took antimalarials before being admitted to the hospital [9]. There were also several studies focusing on the evaluation of antimalarial therapeutic efficacy in uncomplicated *Plasmodium falciparum *cases [10-12].

A standard study protocol for Rapid Urban Malaria Appraisal (RUMA) was developed in June, 2002 based on a WHO proposal and an Environmental Health Project draft protocol [13,14]. RUMAs were commissioned by Roll Back Malaria (RBM) Partnership for three francophone countries (Côte d'Ivoire, Burkina Faso and Benin) and one anglophone country (Tanzania). The RUMA included: a literature review, the collection of health statistics, a school parasitaemia survey, a health facility survey, malaria risk mapping and a brief review of the health care system. Each of the four assessments provided an overview of the urbanization history, an estimate of the fractions of malaria-attributable fevers, parasite rates for different areas, an outline of health care services, and highlights of the "lessons learned" from the survey. A separate overview considers this work in a wider context [15].

This paper is the third in a series of four country assessment papers. Due to political troubles and resulting security concerns in Abidjan from September, 2002 onwards, the breeding site and health facility mapping activities, as well as the school parasitaemia surveys were interrupted. Hence, the study in Yopougon could only collect data on the rate of reported malaria cases through routine statistics and on the fractions of malaria-attributable fevers in health facilities. This is why the data presented are limited as compared to those from other cities.

## Methods

### Study sites and sample selection

**Figure 1:**
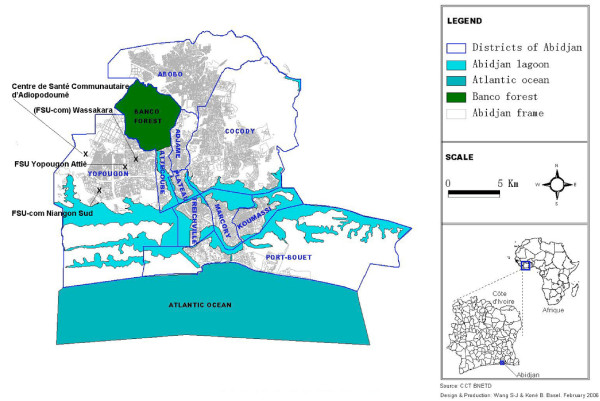
Map of Greater Abidjan with its municipalities and selected health facilities.

Abidjan is situated between latitude 3.7°–4.0° N and longitude 5.7°–6.0° E, with a surface area of 261 sq. km. It is divided into five districts: Central, East, North, South and West (Table 1). The districts are divided into 10 municipalities: Abobo (Abidjan North), Adjamé, Attécoubé and Plateau (Abidjan Central), Cocody (Abidjan East), Koumassi, Marcory, Port-Bouét, Treichville (Abidjan South) and Yopougon (Abidjan West) (Figure 1).

**Table 1:** Demographic information and annual malaria cases reported in Abidjan in 2001. Source: Programme National de Lutte Contre le Paludisme. CHU: Centre Hospitalier Universitaire.

**Districts or CHU**	**Municipality**	**Population**	**Area in km^2^**	**Reported malaria cases**
CHU-Cocody		-		2,525
CHU-Treichville		-		12,375
CHU-Yopougon		-		-

Abidjan-North	Abobo	717,930	112.70	71,437
Abidjan-Central	Adjamé		12.10	
	Attécoubé	519,548	38.60	35,714
Abidjan-East	Cocody	283,174	76.10	55,500
Abidjan-South 1	Plateau	11,659	4.0	-
Abidjan-South 2	Marcory		12.60	
	Treichville	335,518	8.90	-
Abidjan-South 3	Port-Bouét		60.50	
	Koumassi	595,301	11.40	62,607
Abidjan-West	Yopougon	774,171	117.00	-

	**Total**	**3,237,300**	**453.90**	**237,633**

Yopougon is the largest and most recent municipality (117 sq. km) of Greater Abidjan (Figure 1). It comprises both urban and peri-urban areas. The population in Yopougon has grown very fast, from 564 habitants in 1955 to 112,700 in 1975 [16]. In 1998 it comprised one quarter of the total Abidjan population: 774,200 people (density: 5,900 per sq. km). The population is young (50% of the total population is less than 20 years old) and of great ethnic diversity [17].

### RUMA methodology

Details for the RUMA methods are given in an overview publication [15]. As explained above, there was a military conflict in the city centre and intermediate areas of Abidjan since September, 2002 and there was movement control by the military. As a result, the planned mapping activities and school surveys could not be carried out. Briefly, the following components were included in Abidjan.

#### Review of literature and collection of health statistics

Unpublished data on malaria were collected from the Pasteur Institute of Ivory Coast and the National Institute of Public Health. Published information on malaria epidemiology was reviewed systematically through a literature search in the main bibliographic databases (PUBMED and EMBASE), through scanning reference lists and through contacting relevant experts, nationally and internationally. Available demographic and health system information, as well as routine malaria reports, were collected from the Ministry of Health (MOH), the Direction Régionale de la Santé Publique et des Affaires Sociales, the Institut National de la Statistique and the Programme National de Lutte Contre le Paludisme (PNLP) in Abidjan.

#### Health facility fever surveys

The study was carried out September 9–27, 2002. The 27 sectors in Yopougon were classified into three different zones (centre, intermediate and periphery), according to their population density, distance to the centre and physical characteristics. Due to the time constraints of a RUMA, only one representative health facility with a sufficient patient number could be selected in each zone (Figure 1).

##### Centre

Formation Sanitaire Urbaine à base Communautaire (FSU-com) Wassakara, a commercial centre in the sector Gare-Sud Sodeci-GFCI, located at a traffic junction to downtown Abidjan.

##### Intermediate zone

Formation Sanitaire Urbaine (FSU) Yopougon Attié, also called Protection Maternelle et Infantile (PMI), the biggest FSU and the only hospital specialised in maternal and child care in Yopougon. It is situated in the geographical centre of Yopougon, and has an average of 200 outpatients per day.

##### Periphery

FSU-com Niangon Sud, also called Ageforsi, is located beside the lagoon. Unfortunately, the health centres were all shut down from September 19, 2002 onwards, due to an attempted coup and military actions. As a result, it was impossible to recruit a sufficient number of patients in Niangon Sud. A fourth health centre, which was still accessible, had thus to be selected: Centre de Santé Communautaire d'Adiopodoumé, located in the periphery sector KM 17 in the west end of Yopougon.

The study aimed to recruit 200 fever cases and 200 non-fever controls from each health facility, with half of the patients being less than five years old. The following criteria were used for a case definition: outpatients with a history of fever (past 36 hours) or a measured axillary temperature of ≥ 37.5°C. The control group consisted of patients without current or past fever recruited from another department of the same health facility. Controls were matched by age and residency with the fever cases. Participation was voluntary and parents were required to sign a consent form. After giving written consent, questionnaires were administered enquiring about demographic data, socio-economic status and the malaria history for each survey participant. An axillary temperature measure was taken and both thin and thick blood films were prepared on the same slide. Parasite density was defined as the number of parasites per 200 white blood cells. In the end, less than 300 cases and controls could be recruited from each health facility since people were afraid of violence in the street and attendance had fallen sharply.

The odds ratio (OR) that was calculated is the ratio of the odds of having parasitaemia in fever cases over non-fever controls. The formula for the fraction of fever episodes attributable to malaria parasites that was used is the following: (1-1/Odds Ratio)*P, with P being the proportion of fever episodes in which the subjects had malaria parasites [15].

The samples were read at the Centre Suisse de Recherches Scientifiques (CSRS) in Abidjan. For quality control, 133 slides were re-examined at the Swiss Tropical Institute (STI) in Basel. For these 133 slides, 116 readings were accurate. The sensitivity, specificity and accuracy rate were found to be 87.9%, 89.2% and 88.79%, respectively, which was considered acceptable.

#### Brief description of the health care system

Senior officers of PNLP and INS provided essential information regarding the distribution and number of existing providers for malaria diagnosis and treatment in Abidjan. A meeting with representatives of the CSRS and PNLP was held to review the organizational structure of the PNLP, to clarify the national policy and implementation of malaria control, as well as to review the history of resistance to antimalarials.

### Statistical methods

The data were double-entered and validated in EpiInfo 6.04 (CDC Atlanta, USA, 2001). Data analysis was carried out in Stata 8 (Stata Corp. Texas, USA, 2003). The X^2 ^test was applied to assess associations between categorical variables. Logistic regression was performed to assess the association between binary outcomes and explanatory variables.

### Ethics

The study received ethical clearance from the Ivorian national ethics committee. All the patients gave informed consent. A prescription of chloroquine or amodiaquine was paid for if the patients presented fever signs or had parasitaemia.

## Results

### A brief description of the health care system

The public health care system in Abidjan consists of a number of different units. The Centre Hospitalier Universitaire (CHU) functions as the first-level referral and teaching hospital (three CHU in Abidjan: CHU-Treichville, CHU-Cocody and CHU-Yopougon).

The two most populated municipalities of Abidjan, Yopougon and Abobo, had striking deficiencies in health care delivery. In 1992 there were only two primary health care centres, one serving approximately 530,000 people in Abobo and the other serving 540,000 people in Yopougon [18]. At the end of 1997, 11 Formation Sanitaire Urbaine à base Communautaire (FSU-com) had been established and a new pricing policy for the Formation Sanitaire Urbaine (FSU) and FSU-com were introduced to help low-income families [19,20].

**Figure 2:**
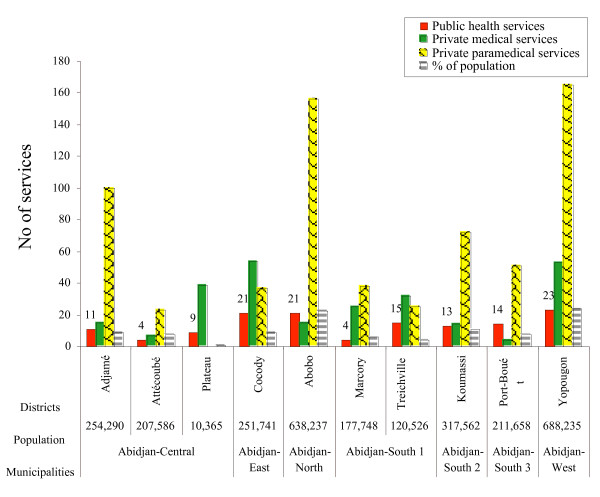
Distribution of public, private and paramedical facilities in Greater Abidjan, 2001.

By the end of 2002 there were 135 public and 238 private medical services in the greater Abidjan (Figure 2), as well as 667 private paramedical services, usually run by nurses and specialised in gynaecology, paediatrics or internal medicine. The distribution of private paramedical services in the different municipalities was heterogeneous. There were 165 paramedical services in Yopougon and 156 in Abobo, nearly 40% of all Abidjan.

One public health centre serves an average of 21,300 inhabitants in Abidjan. Not surprisingly, better-off people benefit from more health care resources despite the recent health system reform. Plateau, being the smallest and richest municipality in Abidjan, has the highest coverage of public and private health services. In 1998 each public health centre served only 1,200 inhabitants, and each private health facility served only 270 inhabitants there. By contrast, there were 30,400 and 51,900 inhabitants for one public health centre in Yopougon and Attécoubé.

The Programme National de Lutte Contre le Paludisme (PNLP) was started in 1992 [16,21] and Roll Back Malaria (RBM) strategies were introduced in January, 2001: case management, chemoprophylaxis for pregnant women, individual and household protection against vectors, reinforcing the health care system, community activities and building up the RBM Partnership. Unfortunately, none of these strategies have been implemented on a large scale.

### Results of malaria routine reports

The estimation of malaria incidence rates was based on the routine data collected by the PNLP in 2001. Reporting of malaria is neither systematic nor consistent in Abidjan [21]. In 2001 the data from CHU-Yopougon and Yopougon and Plateau municipalities were missing (Table 1). The malaria cases reported from CHU-Cocody and CHU-Treichville were separated from the other data since these teaching hospitals had patients referred from the countryside and from other health centres in Abidjan. Malaria cases from the CHUs are, therefore, not representative of the area in which they are located. In 2001, there were around 600,000 consultations for all causes in public health facilities, and roughly 240,000 cases (40.9%) were due to malaria. Very few cases (5.2%) were laboratory confirmed.

**Figure 3:**
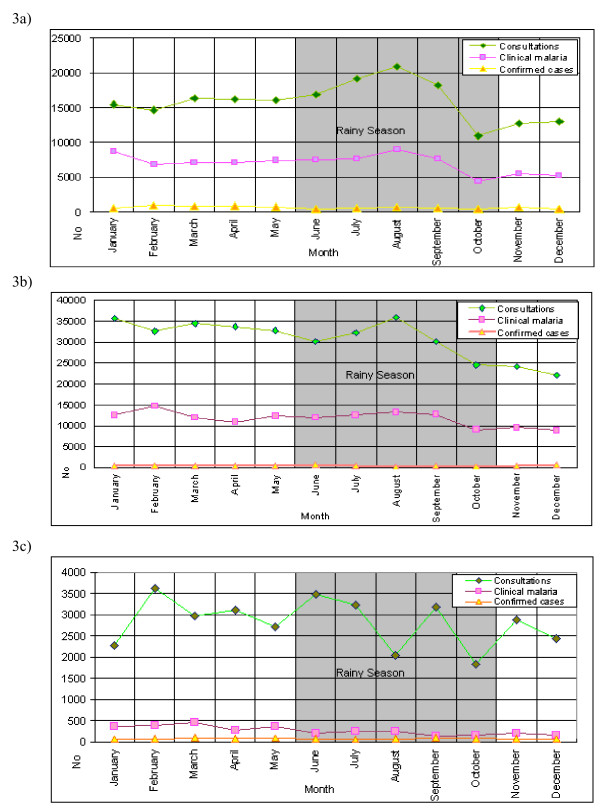
Numbers of overall consultations, suspected and confirmed malaria cases in Abidjan, 2001. 3a) children under five years; 3b) persons over five years; 3c) pregnant women.

There are three seasons in Abidjan, (1) the hot and wet season from June to October, (2) the warm and dry season from November to March and (3) the hot and dry season from March to May. Maximum rainfall usually occurs between August and September. However, seasonality of malaria in the dataset was minimal and only a slightly lower number of cases was registered in October-December. Figure 3 shows the number of reported total consultations (all causes), reported and confirmed malaria cases by month for children under five years of age (3a), persons over five years of age (3b) and pregnant women (3c).

### Health facility-based surveys

*P. falciparum *was detected in 26.1% of all blood slides. A total of 149 (34.7%) of 429 fever cases and 63 (16.4%) of 383 controls were malaria positive. The participants were stratified into four age groups: under one year old, one to five years old, > five to 15 years old, and adults >15 years old. The percentages of parasites detected in febrile cases were 23.1%, 43.0%, 43.8% and 25.6% for these age groups, while the parasitaemia rates in the controls were 13.0%. 26.7%, 22.9% and 14.3% (Table 2). The difference between age groups was statistically significant (P < 0.05). In the 1–15 years old group over 40% of fever cases and only 22%–26% of the controls were parasitaemic. The overall malaria prevalence in infants ≤six months was 14.5% and among them 28% had a hyperparasitaemia of more than 6,400/μl.

**Table 2:** Malaria infection rates in fever cases and controls in Yopougon, as well as odds ratio for parasitaemia, by age groups. Health facility-based surveys.

	**Fever cases**	**Controls**	**OR**	**95% CI**	**P value**	**Fractions of malaria-attributable fevers**
Age groups	N = 429	N = 383				
Infants 0–1 year	18/78 (23.1%)	22/169 (13.0%)	2.00	0.95–4.22	<0.05	0.12
Children 1–5 years	61/142 (43.0%)	16/60 (26.7%)	2.07	1.02–4.24	<0.05	0.22
Children 6–15 years	39/89 (43.8%)	8/35 (22.9%)	2.63	1.00–7.11	0.05	0.27
Adults >15 years	31/120 (25.6%)	17/119 (14.3%)	2.09	1.04–4.25	<0.01	0.13

The risk of having parasitaemia in fever cases was significantly higher than for the control group, with odds ratios (OR) of 2.00, 2.07, 2.63 and 2.09 for the above age groups. As a result, the fractions of malaria-attributable fevers were 0.12, 0.22, 0.27 and 0.13, respectively, suggesting that malaria played a low to moderate role in fever episodes during this season (Table 2).

Parasitaemia was more frequently found associated with a high fever. For children under five years, the OR of having parasitaemia with a fever ≥39°C that lasted two to four days was 3.35 (95% CI = 1.36–8.32, P < 0.005) in comparison to a fever <39°C of the same duration (Table 3). The OR of having malaria parasites with a fever ≥39°C lasting for five to seven days was 6.8 times greater than having a fever <39°C of the same duration (95% CI = 1.32–38.95, P < 0.01) (Table 3).

**Table 3:** Fever intensity and risk of parasitaemia in Yopougon. Health facility-based surveys.

**All age groups**	**Fever intensity**	**Positive/Total (%)**	**OR**	**95% CI**	**P value**
Fever duration	<39°C	71/215 (33.0%)	1	-	-
≥2, <5 days	≥39°C	26/46 (56.5%)	2.64	1.32–5.30	<0.005
Fever duration	<39°C	26/100 (26.0%)	1	-	-
≥5, <8 days	≥39°C	11/16 (68.8%)	6.26	1.78–23.17	<0.001
**≤5 years-old**	**Fever intensity**	**Positive/Total (%)**	**OR**	**95% CI**	**P value**
Fever duration	<39°C	35/109 (32.1%)	1	-	-
≥2, <5 days	≥39°C	19/41 (46.4%)	3.35	1.36–8.32	<0.005
Fever duration	<39°C	13/46 (28.3%)	1	-	-
≥5, <8 days	≥39°C	8/11 (72.7%)	6.77	1.32–38.95	<0.01

### Socio-economic factors

**Figure 4:**
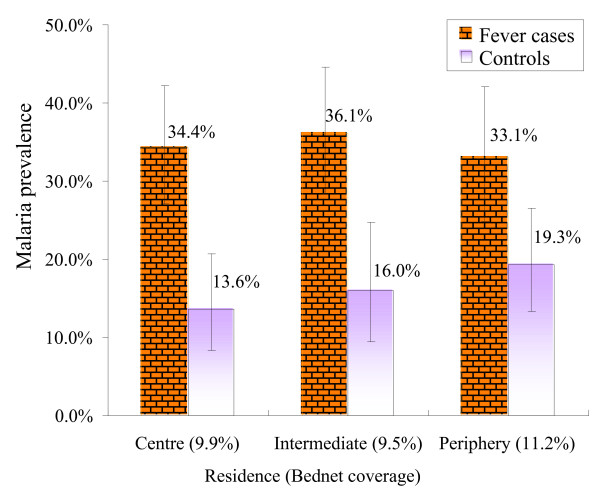
Malaria prevalence detected in fever cases and non-fever controls in Yopougon, by residence of patients. Health facility-based surveys. Vertical bars represent 95% CI.

Malaria infection rates were fairly similar in fever cases across the three residential zones and the same was true for non-fever controls (Figure 4). Further, a logistic regression model was used to assess the association between parasite infections and the different zones. The OR was 1.27 (95% CI = 0.86–1.86) in the intermediate zone and 1.17 in the periphery (95% CI = 0.80–1.81) compared to the centre (Table 4).

**Table 4:** Socio-economic factors and the risk of malaria infection in fever cases and controls in Yopougon. Logistic regression model. Health facility-based surveys. N = 812.

**Socio-economic factors**	**% of total**	**OR**	**95% CI**	**P value**
**Education level**				
Primary	29.4%	1	-	-
**Secondary**	**26.7%**	**0.54**	**0.35–0.84**	**<0.01**
**Superior**	**8.7%**	**0.37**	**0.18–0.77**	**<0.01**
Other	35.2%	0.76	0.51–1.12	0.17
**Housing material**				
Leaf/mud	7.7%	1	-	-
Concrete/brick	91.7%	0.65	0.35–1.2	0.1
**Water supply resource**				
Well	1.4%	1	-	-
Tap water	95.6%	1.67	0.34–11.14	0.74
Fountain	2.0%	1.07	0.11–11.54	1.00
**Living nearby agriculture land or gardens**				
No	20.9%	1	-	-
Yes	75.4%	1.05	0.70–1.59	0.8
**Bednet usage (treated and untreated)**				
No	89.9%	1	-	-
**Yes**	**10.1%**	**0.52**	**0.29–0.97**	**<0.05**
**Rural exposure within 90 days**				
No	68.9%	1	-	-
**Yes**	**31.1%**	**1.75**	**1.25–2.45**	**<0.001**
**Previous malaria treatment within 30 days with the presence of parasitaemia**				
No	63.3%	1	-	-
Yes	36.7%	1.09	0.79–1.53	0.6
**Residence of patients**				
Centre	35.3%	1	-	-
Intermediate	30.2%	1.27	0.86–1.86	0.2
Periphery	34.5%	1.17	0.80–1.72	0.4

The percentage of patients sleeping under a bednet on the night before admission to the hospital was low at 10.1%, with only 6.9% of the total study population having ITNs. Usage rates were low and similar in the three zones (9.9%, 9.5% and 11.2%, Figure 4). While infrequent, the use of bednets had a strong protective effect against a malaria infection in Yopougon (OR = 0.52, 95% CI = 0.29–0.97).

The OR of having a malaria infection was lower if the patients or caretakers had a higher education level (secondary schools: 0.54, 95% CI = 0.35–0.84; college and above: 0.37, 95% CI = 0.18–0.77)(Table 4). There was no evidence of association between housing type, water supply sources and proximity to urban agriculture land with a malaria infection.

Travelling to rural areas within the last three months was a risk factor for being infected with *P. falciparum *(OR = 1.75, 95% CI: 1.25–2.45). In Yopougon, 38.2% of patients with parasitaemia had been outside Abidjan within the last three months. There was no association between previous malaria treatment and the current presence of parasitaemia (Table 4). In total 304 respondents claimed to have had a malaria infection within 30 days of the survey; of those, 65.7% reported having been treated in health facilities and about 31.9% used an informal therapy: they went to pharmacy/drug outlets, used traditional healers, self-treated at home or underwent no treatment.

## Discussion and conclusion

One of the limitations of this assessment was that the study was done in only one municipality and results are, therefore, not representative for the whole city (with the exception of the routine statistics, which were compiled for all municipalities). In future, an in-depth assessment in the other municipalities of Abidjan would help to describe more comprehensively malaria patterns in this large city and help resolve the question of whether the malaria risk is similar everywhere. Given the steep socio-economic gradient within the city this is very unlikely, but it needs to be described.

Remarkably, there was almost no seasonality in reported clinical malaria cases in Abidjan in 2001. This was unexpected and does not fit observations from other cities in SSA, especially recent reports for similar settings in Cotonou and Dar es Salaam [15]. However, the accuracy of PNLP routine malaria report is uncertain. In addition, there is a high rate of malaria over-diagnosis, as evidenced by the low-to-moderate malaria-attributable fractions (see below), If most fever cases are not due to malaria, this might explain at least in part why so called "malaria" cases present with such regularity over the year.

The RUMA methodology is based on a cross-sectional design during a single season. Hence, the results of the parasitaemia survey may be different at another time of the year, especially during the dry season (November to May). As a result, it was not possible to cross-check the pattern of malaria cases reported through routine health services within the frame of this study. Repeating this work at a different season might shed some interesting light on transmission patterns.

Unfortunately, a good part of the planned RUMA activities could not be accomplished due to the political instability (the school parasitaemia surveys and the mapping of *Anopheles *breeding sites). School parasitaemia surveys in the same zones would have given a more representative (community-based) measure of parasitaemia in Yopougon and would, therefore, have better defined local endemicity. It would also have helped to cross-check the results from the health facility fever surveys. The present results from the control group in the health facilities surveys suggest that endemicity is moderate, ranging from 13 to 27% for the different age groups. The distribution by the different zones (range: 14–19%) did not show much variability. This finding was rather unexpected since Yopougon borders on more rural areas and one would have expected parasite prevalence rates to be higher at the periphery. It is possible that since the whole of Yopougon is located along the major roads to downtown Abidjan, the heterogeneity of transmission was reduced. Another reason could be that the sample population attending FSU-com was homogenous in their socio-economic characteristics and hence in their malaria risk. Based on unpublished results of the Abidjan Health Project (*Projet Santé Abidjan*), the patients attending FSU-com and CSU were similar in terms of their income and education level [22].

An important finding was that the fractions of malaria-attributable fevers in health facilities were low to moderate in all age groups during the time of the present survey. Between 73 and 88% of all fever cases presenting at health facilities are unlikely to have suffered from malaria. This has two main implications for the health system. Firstly, there is substantial over-treatment with antimalarials, which exposes patients to unnecessary side-effects and wastes precious resources. Secondly, other potentially dangerous conditions might be missed. It is not clear whether this high level of over-treatment is constant throughout the year in Abidjan, but it is certainly consistent with three other studies carried out recently in SSA [15].

In high transmission areas, children under the age of five years bear the highest risk for malaria. In this study, malaria infections and hyperparasitaemia were not rare in infants and simultaneously there were higher infection rates in elder children. This might suggest a mixed or transitional pattern of high/low malaria transmission levels in Yopougon.

In conclusion, the malaria seasonality and endemicity in different areas of Greater Abidjan needs to be further assessed. Given the low to moderate attributable fractions of malaria in febrile episodes and the resulting high rate of misdiagnosis, health facilities should consider introducing a proper diagnosis of "malaria cases", possibly through the use of rapid tests.

## Authors' contributions

SW participated in the design of the study, conducted the field work, analysed and interpreted the data, drafted and revised the manuscript. CL conceived the study, coordinated the field work and revised the manuscript. TS and PV participated in the design and statistical analysis. GC was the key contact person in the field, managed and supervised the data collection and laboratory work. MT participated in the conception of the work, revised it critically at all stages and contributed to the writing of the manuscript.
